# The Impact of ART on the Economic Outcomes of People Living with HIV/AIDS

**DOI:** 10.1155/2013/362972

**Published:** 2013-01-28

**Authors:** Annet Nannungi, Glenn Wagner, Bonnie Ghosh-Dastidar

**Affiliations:** ^1^Infectious Diseases Institute Makerere University, Kampala, Uganda; ^2^Rand Corporation, Santa Monica, CA 90401, USA

## Abstract

*Background*. Clinical benefits of ART are well documented, but less is known about its effects on economic outcomes such as work status and income in sub-Saharan Africa. *Methods*. Data were examined from 482 adult clients entering HIV care (257 starting ART; 225 not yet eligible for ART) in Kampala, Uganda. Self-reported data on work status and income were assessed at baseline, months 6 and 12. Multivariate analysis examined the effects of ART over time, controlling for change in physical health functioning and baseline covariates. *Results*. Fewer ART patients worked at baseline compared to non-ART patients (25.5% versus 34.2%); 48.8% of those not working at baseline were now working at month 6, and 50% at month 12, with similar improvement in both the ART and non-ART groups. However, multivariate analysis revealed that the ART group experienced greater improvement over time. Average weekly income did not differ between the groups at baseline nor change significantly over time, among those who were working; being male gender and having any secondary education were predictive of higher income. *Conclusions*. ART was associated with greater improvement in work status, even after controlling for change in physical health functioning, suggesting other factors associated with ART may influence work.

## 1. Introduction

Since 2003, with the support from international initiatives such as the “3 × 5” campaign of the WHO, UNAIDS, and Global fund to fight AIDS, TB, and Malaria which sought to have 3 million on antiretroviral therapy (ART) by end of 2005 [1] and funding from the US government's President's Emergency Plan for AIDS Relief (PEPfAR), there has been a wide scale-up of ART in the region [2, 3]. Clinical effectiveness and biomedical benefits of ART have been well documented [4–7]. However, much less research has examined the extent to which these benefits translate into socioeconomic well-being for individuals and their households. One might expect that improved physical health and functioning from ART might consequently result in increased work, productivity, and labour force participation. It's upon this hypothesis that this study examined the effects of ART on work and income levels among HIV clients in Uganda. 

Beard et al. [8] published a systematic review of research examining the effects of ART on economic well-being for individuals in resource constrained settings. Findings revealed that compared to patients not yet on ART, work performance improved and absenteeism decreased among patients on ART, with these changes taking place in the first 3 months of treatment and then leveling off. Since this review a study was published from Kenya [9] that found that the effects of ART on productivity and absenteeism among tea farm workers interacted with gender. Study results indicated that there was greater impairment in work productivity (number of days spent plucking tea) in women compared to men prior to the start of ART and throughout the first year of ART. The study compared HIV-infected tea estate workers and a comparison group of HIV-negative workers assigned to the same work teams. After one year on ART, the HIV-positive women worked 30% fewer days plucking tea per month and about 100% more days on nonplucking tasks, compared to the comparison group. In contrast, men on ART were able to maintain a similar pattern of work as their uninfected counterparts except during the initial five months of therapy. The authors proposed that a possible explanation for this gender difference is greater household demands on the time of women, which may translate into increased fatigue and poorer work functioning. 

The studies reviewed above focused on people who had jobs in the formal labour market and therefore results pertained to work absenteeism and productivity, not on whether or not someone was able to engage in work or find a job. In a small qualitative study conducted in Uganda, Wagner et al. [10] found that loss of work after HIV diagnosis was common, but after starting ART patients were able to engage in some type of work, though often not at the same level as before HIV. The authors concluded that as much as ART may help people to regain capacity to work, other economic supports are needed to enable individuals and households to reestablish their livelihoods, especially in resource-constrained settings. In one of the few quantitative studies to examine the effects of ART on work status, Thirumurthy and colleagues [11] recently published results that indicated ART resulted in a rapid and sustained increase in employment and income for patients in India. The results indicated that 6 months after initiation of ART, patients were 10% more likely to be economically active, compared to the control group of HIV-infected individuals not on ART. Similar to the finding of Larson et al. [9], results indicated that effects were almost twice as large for men compared with women. 

This paper reports analyses from a prospective observational cohort study conducted in Uganda to study the effects of ART on multiple health outcomes, including economic well-being. Analyses were conducted to address the questions of whether patients on ART would differentially benefit from treatment compared to nonART patients with regard to work status and income. A greater understanding of the economic benefits of ART could inform policy regarding the importance of sustaining the availability of ART for people living with HIV/AIDS. 

## 2. Methods

### 2.1. Sample

The study was conducted at two HIV clinics, Reachout Mbuya and Mulago ISS (Immune Suppression Syndrome) Clinic, both in Kampala, the Ugandan capital. Between July 2008 and August 2009, consecutive new clinic clients who had just completed evaluation for ART eligibility were enrolled in a longitudinal cohort study designed to examine the impact of ART on social and economic well-being. The primary criterion for ART eligibility was having either a CD4 count ≤ 250 cells/mm or WHO stage 3 or 4 disease (AIDS diagnosis). Those who were not yet eligible for ART were required to have a CD4 count of less than 400 cells/mm^3^. All participants received HIV primary medical care, which includes monitoring and treatment of active infections, and prescription of appropriate prophylactic medications. Assessments were made at study enrollment, months 6 and 12 for all participants; however, nonART patients who started ART during the course of the study were administered the assessment at ART initiation and then 6 months later. Participants were paid 6000 Uganda Shillings ($2.50) after each assessment. The protocol was approved by the Institutional Review Board of Makerere University, College of Health Sciences and the Uganda National Council of Science and Technology. 

### 2.2. Measures

The primary outcomes of the analysis were work status and weekly income. The variables examined as potential determinants of these outcomes included demographics and measures of physical health. All variables were measured at each assessment.

#### 2.2.1. Work Status

Respondents were asked whether they engaged in work activity in the last 7 days, with a binary response of yes or no. Such work activities could include jobs where they are paid in cash or in kind, selling things, running a small business, or working on the family farm or in the family business. 

#### 2.2.2. Weekly Income Level

Participants were askedhow much their last payment was and for what number of weeks this payment covered. Thereafter a weekly mean income was computed. 

#### 2.2.3. Impact of HIV and Health Status on Work and Income

At baseline participants were asked how their work and income levels had changed since they tested HIV positive. Participants were asked how often their health had affected their ability to work over the past month, with response options being “never,” “rarely,” “sometimes,” and “most of the time.” Also respondents were asked if their income was better, worse, or similar since HIV diagnosis, and whether they had to stop or cut down on the work they used to do since the diagnosis. 

#### 2.2.4. Demographic and Background Characteristics

These include gender, education levels (primary school or less versus having at least some secondary school), age, and relationship status (married or in a committed relationship versus being single, divorced, or widowed). 

#### 2.2.5. Physical Health

CD4 count at the time of entry into care was abstracted manually from the patient's medical chart. Physical health functioning was measured with the 6-item subscale of the *Medical Outcomes Study HIV Health Survey*, [12] which assesses the extent to which the respondent feels limited by their health in being able to engage in a range of daily activities (e.g., lifting heavy objects; moving a table or carrying groceries; walking one block; eating, dressing, bathing, or using toilet); response options include “yes, limited a lot,” “yes, limited a little,” and “no, not limited at all,” and the subscale score is standardized on a 0–100 scale, with a higher score representing better functioning. 

### 2.3. Data Analysis

Bivariate statistics (two-tailed *t*-tests, chi square tests) were used to compare baseline characteristics across the ART and nonART groups, and to examine (unadjusted) within-group change over time (paired *t*-test, McNemar's test). A staged approach to performing multivariate longitudinal regression models was used to examine the effects of ART on outcomes measured across the three assessments, and the potential explanatory role of change in physical health functioning. In these models we used the generalized estimating equation method for analysis of correlated data to model the repeated measurements, assuming a normal distribution for the weekly mean income variables, and a binomial distribution for the dichotomous outcome of work status. As an initial step, we analyzed models to examine change in the dependent variable (work status and weekly income in separate models) across the three study assessments, and the independent variables included ART status (representing whether or not there is a group difference in the dependent variable at baseline), time (ordinal variable representing the change in the dependent variable for each additional unit of time (i.e., 6 months) over the three time periods, which is attributed to HIV medical care), and the interaction of ART status by time (represents the additional change in the dependent variable with each additional unit of time among patients in the ART group relative to the group receiving HIV medical care alone or the nonART group). Covariates that were added to the model included patient characteristics that controlled for differences in the ART and nonART groups at baseline (CD4, physical health functioning) as well as age, education level, relationship status, and gender. Analyses included attrition weights to account for dropout; these were derived using logistic regression with study completion status as the outcome and baseline measures associated with ART and completion status as independent variables. In the second step, the same models were reexamined but with the addition of change in physical health functioning from baseline to month 12, in separate analyses.

#### 2.3.1. Sensitivity Analyses

The primary analysis used an intention-to-treat (ITT) approach, which included all participants in the ART and nonART groups at baseline, thus resulting in a conservative estimate of the effects of ART given that some nonART patients (*n* = 24) started ART during the study period. We augmented the ITT analysis with a sensitivity analysis in which we excluded the 24 nonART patients who started ART during the course of the study. The sensitivity analysis was performed for each of the two primary outcomes. In each case, the findings remained unchanged from the original models; therefore, we have chosen not to present the data from the sensitivity analysis. 

## 3. Results

### 3.1. Sample Characteristics

The sample consisted of 482 participants, of whom 257 (53.0%) were starting ART and 225 (47.0%) were not yet eligible for ART; 36.1% were males. The demographic characteristics (age, gender, education, and relationship status) as well as health status (CD4 count and physical health functioning) of the total sample, as well as separately for ART and nonART participants, are listed in Table 1. The ART group differed from their nonART counterparts with regard to physical health functioning and CD4 cell count, with the ART groups having lower CD4 count and physical health functioning. 

### 3.2. Impact of HIV and Health Status on Work and Income

At baseline, nearly half (47.2%) reported worse income since testing HIV positive, but roughly the same proportion of participants (44.1%) reported having similar income, while 8.7% reported having better income. When asked about the effect of their current health status on work performance, 37.0% reported that their health over the past month “rarely” or “never” affected their ability to perform at work, whereas 40.4% said it did “some” or “most” of the time, and 3.9% reported being unable to work at all because of their health. After testing HIV positive, 54.0% continued to work, 21.5% could still work but not as much as they used to or had to change to work that was less physically demanding, 15.9% stopped working, and 8.7% were not working before and were still not.

### 3.3. Baseline Measures of the Primary Outcomes (Work Status, Income)

At baseline, 69.5% reported working during the last 7 days, of whom 30.5% reported being self-employed and 72.8% were employees of others. ART patients were less likely to be working at baseline compared to nonART patients (34.2% versus 25.5%, *P* = .041). Average weekly income at baseline was 32,356 (SD = 28,277, median = 25,000) for ART group, which was similar to the mean of 32,879 (SD = 58,267, median = 21,000) for the nonART group (*P* = .278). 

### 3.4. Longitudinal Analysis of Change in the Primary Outcomes

Attrition was high at month 6, with 30.0% of the total sample not completing assessments, but then leveled off with only a slight increase to 36.0% not completing assessments at month 12. When examining attrition across groups, the ART group had a higher rate of noncompletion at month 6 (35.0%) compared to the nonART group (24.0%; *P* = .006), but at month 12, the two groups were similar with 37.0% of the ART group not completing the assessment compared to 35.0% of the nonART group. The completers and dropouts did not differ on any demographic or illness related characteristics at baseline. 

### 3.5. Change in Work Status

While a higher proportion of the nonART group was working at baseline, as reported above, the proportion currently working did not differ between the ART and nonART groups at month 6 (53.0% versus 47.0%, *P* = .37) or month 12 (46.0% versus 54.0%, *P* = .18). However, examining change over time among those who were working and those who were not working at baseline, separately, revealed a significant pattern of change. Of the ART respondents who were working at baseline, the vast majority continued to be working at month 6 (81.4%, *P* = .000) and month 12 (87.7%, *P* = .000) among those who remained in the study at the follow-up assessments; in contrast, there was considerable change among those not working at baseline, with half (50.9%) reporting to be working at month 6, and 55.6% at month 12. Findings were very similar in the nonART group; of those who were working at baseline, 84.5% were working at month 6 (*P* = .000) and 75.9% at month 12 (*P* = .004) among those who remained in the study at the follow-up assessments, while 48.8% of those not working at baseline were now working at month 6, and 50.0% were working at month 12.

The multivariate ITT analysis of work status over the three study assessments revealed a difference between the ART and nonART groups at baseline, with more of the ART group not working as compared to the nonART group. The overall time trend for the group as a whole was insignificant; however, the interaction of time and ART status was significant, indicating that the improvement in work status in the ART group was significantly greater than that in the nonART group (see Model 1 in Table 2). Among the baseline covariates that predicted improved work status over time was male gender, better physical health functioning, and higher CD4 count. When change in physical health functioning was added to the model, change in physical health functioning was a strong predictor, with improved physical health functioning being associated with improved work status (see Model 2 in Table 2). All the variables that were significant predictors of work status in the initial model continued to be significant. 

### 3.6. Change in Weekly Income

Bivariate paired *t*-tests were used to compare the baseline mean income levels and the income levels at 6 and 12 months among those who remained in the study at the follow-up assessments. The ART group experienced a reduction in weekly income from a mean of 33,775 Ush at baseline to 29,481 Ush at month 6, which was marginally significant (*P* = .08), but then increased to an average weekly income of 38,516 Ush at month 12; this change from month 6 to 12 was also marginally significant (*P* = .08), but the income level at month 12 was now back to an equivalent level as that at baseline (*P* = .81). In the nonART group, a similar pattern was observed with a reduction in weekly income from a mean of 31,034 Ush at baseline to 25,909 Ush at month 6, which was not statistically significant (*P* = .18), followed by a significant increase to an average weekly income of 36,550 Ugs at month 12 (*P* = .006). Just as at baseline where the ART and nonART groups had equivalent levels of income, there were no significant differences in income between the two groups at month 6 (ART mean = 28,949, nonART mean = 25,915; *P* = .28) and month 12 (ART mean = 37,268, nonART mean = 35,257; *P* = .64). 

In the multivariate ITT models of income levels (see Model 1 in Table 3), the ART and nonART groups did not differ at baseline, the ART and nonART groups did not differ in their experience of a change in income over time, and there was no significant change in income in the sample as a whole. Among the covariates in the model, male gender and having any secondary education were predictive of an increase in income over the first year of treatment, and these two variables continued to be the only predictors of income when change in physical health functioning was added to the model (see model 2 in Table 3). 

## 4. Discussion

HIV has a significant negative impact on the work and income of PLHA, and this was true for many in our sample as well, and while our findings suggest that HIV care in general, and ART even more so, is often associated with a greater likelihood of working, this benefit does not necessarily translate into increased income. While HIV care and ART appeared to enable people who were not working at baseline to find and be able to work within the first 6 months of treatment, reported levels of income among patients who worked throughout the study remained essentially the same in both ART and nonART patients. 

While many of our participants had lost work because of their HIV disease, most were working at entry into care (study baseline) and continued to work throughout the first 12 months of HIV care. However, it was those who were not working at baseline who experienced the most benefit from treatment. Roughly half of the participants who were not working at baseline reported working at Months 6 and 12. This was true for both ART and nonART patients, but our data did reveal a significant ART effect with regard to work status. At entry into care, those eligible for ART were worse off compared to those not yet eligible for ART as they were less likely to be working and had lower CD4 counts, but over the course of 12 months of care the multivariate analysis revealed that the ART group experienced greater improvement in work status. Similar to the findings reported by Beard et al. [8], the benefits of treatment on work were observed in the first 6 months of treatment, with little or no change thereafter. 

Physical health functioning, a presumed key factor by which ART and HIV care influences work status and income, was significantly associated with change in work status, both in terms of the baseline level of physical health functioning as well as change in physical health functioning over time. However, the fact that the ART group continued to show an advantage in work status over time, even after controlling for change in physical health functioning, suggests that there are other aspects of ART that we have not measured that influence work status. One possible explanation is the greater exposure to social support at the clinic setting (from both providers and patients) for ART versus nonART patients, since ART patients have to go to the clinic monthly (to pick up drug refills) as opposed to every 6 months for nonART patients. This increased support could lead to greater reduction in internalized HIV stigma and increased empowerment and self-efficacy regarding work functioning.

In regard to impact of ART on income, it stayed the same more or less throughout the study for those who were working. There was some reduction in mean income at month 6, but the change was not significant, perhaps as a reflection of the data not being normally distributed and having a large variance. By month 12, mean income levels had returned to a level equivalent to that measured at baseline. It is important to note however that income data were only collected from those who were working. One would assume that the income increased for the subgroup that was not working at baseline but was working at followup, but our data were unable to assess this because income level was only asked of those who were working at the time of the assessment. It is also worth noting that a longer followup, beyond 12 months, may be needed to adequately assess effects on income, as there may be a more lagged effect of treatment on income as opposed to work activity.

Among the covariates that predicted an increase in income were being male gender and having any secondary education. One possible reason for male gender being a predictor of increased income is that females may experience greater challenges with regard to household demands on their time (e.g., child care, household chores, and maintenance) that impede their ability to engage in other forms of work, and which were not categorized as work activity in this study even though such household activities are arguably of similar value to income generating activity. Another possible reason is that in Uganda culture, men are the breadwinners for their families, so men have to try all means to get a source of income, especially those who are married, resulting in men being more motivated to increase income. The positive relationship between income and having any secondary education is not surprising as it suggests that a higher education is associated with greater income earning potential and ability to get higher paying work. 

The study has a number of limitations that are worth noting. With widespread access to ART in Uganda at the time of study enrollment, we could not ethically randomly assign ART to matching groups. This resulted in a less than optimal comparison group, as there were clear indicators that the nonART group had better physical health at baseline than the ART group, although the nonART group did have evidence of immune suppression (CD4 < 400). Also, while the nonART group enabled us to control for time trends in the context of receipt of HIV care, the lack of a comparison group of HIV-infected persons not in HIV care prevented us from being able to account for natural changes in the outcomes that may occur over time in the absence of HIV medical care. Being a longitudinal study, attrition and incomplete data were a challenge as demonstrated by 30% not completing the month 6 interviews; however, there was little added attrition by month 12 and our analyses included attrition weights in order to prevent any bias on the multivariate analyses. Our evaluation of work status is limited by the relatively crude nature of our measure of work status and short time frame (past 7 days); future research should examine a longer time frame and a more nuanced breakdown of work in terms of occupation type, number of hours worked, absenteeism, and productivity level while at work. Our findings cannot be considered generalizable to all persons living with HIV, in particular those not in HIV care or those who have been in care for several years, nor those in more rural settings where job opportunities may be lesser than opportunities for those in Kampala. Lastly, our assessment battery did not include measures of variables such as prior work experience or environmental factors influencing the economic job market, which could influence work status and income.

In conclusion, our findings revealed a significant beneficial effect of ART on work status, compared to nonART clients who had immunosuppression but less advanced HIV disease and were thus healthier as a group overall. Though both the ART and nonART groups experienced improvement in work status, particularly those who were not working at entry into care, improvement in work status over the first year in HIV care was greater for the ART group. This ART advantage was true even after controlling for change in physical health functioning, which suggests that there are other factors associated with ART that may influence work. Our data did not support any noticeable effect of ART or HIV care on income, but this assessment was limited to only those who were working at the onset of HIV care and thus requires further examination to more fully evaluate the effects of HIV treatment on income and overall economic well-being. Lastly, as WHO and country policies move towards starting patients on ART with higher CD4 counts, ART will be started among people who are healthier; therefore, future research will be needed to examine how this may modify the effects of ART on work status and other activities related to physical health functioning.

## Figures and Tables

**Table 1:** Baseline demographic, physical health, and economic characteristics of total sample and by ART status (*N* = 482).

	Total	On-ART	Non-ART	Test statistics
*N *	482	257 (53%)	225 (47%)	
Gender				
Male	36.10%	43.60%	27.60%	*χ* ^2^ = 13.35, *P* = .000
Female	63.90%	56.40%	72.40%	
Mean age	34.6	34.8	34.3	*F* = .001, *P* = .977
Education				
Primary school or less	58.90%	57.00%	61.30%	*χ* ^2^ = .854, *P* = .355
At least some secondary school	41.10%	43.00%	37.70%	
Marital status				
Married/in a committed relationship	48.50%	52.50%	44.00%	*χ* ^2^ = 3.49, *P* = .061
Not in a relationship	51.50%	47.50%	56.00%	
Physical health functioning	71.96	65.32	80.98	*χ* ^2^ = 8.722, *P* = .003
Working status				
Currently working (last 7 days )	69.50%	65.80%	74.50%	*χ* ^2^ = 4.18, *P* = .041
Not working (last 7 days )	30.50%	34.20%	25.50%	
Mean CD4 count	228	142	452	*F* = 4.497, *P* = .000
Mean weekly income (Ush)	32,707	32,356	32,879	*F* = 1.178, *P* = .278

**Table 2:** Multivariate logistic regression examining ART impact on work status.

Variables	OR	95% CI	*P* value
Model 1			

ART	0.48	(0.30, 0.78)	0.00
Time	0.90	(0.72, 1.12)	0.34
ART × time	1.56	(1.15, 2.12)	0.00
Baseline covariates			
Age	1.01	(0.99, 1.04)	0.28
Gender	0.43	(0.29, 0.64)	0.00
Any secondary education	1.35	(0.93, 1.95)	0.12
In relationship	0.76	(0.52, 1.11)	0.15
CD4	1.00	(1.00, 1.00)	0.04
Physical health functioning	1.02	(1.01, 1.02)	0.00

Model 2			

ART	0.49	(0.30, 0.80)	0.00
Time	0.86	(0.68, 1.08)	0.20
ART × time	1.45	(1.06, 1.99)	0.02
Change in physical health functioning	1.01	(1.00, 1.02)	0.01
Baseline covariates			
Age	1.01	(0.99, 1.04)	0.23
Gender	0.44	(0.30, 0.66)	0.00
Any secondary education	1.35	(0.93, 1.95)	0.11
In relationship	0.76	(0.52, 1.11)	0.15
CD4	1.00	(1.00, 1.00)	0.03
Physical health functioning	1.02	(1.01, 1.03)	0.00

**Table 3:** Multivariate linear regression examining ART impact on weekly income.

Variables	Beta	SE	*P* value
Model 1			

ART	−6105	4036	0.13
Time	1421	1959	0.47
ART × time	−280	2852	0.92
Baseline covariates			
Age	92	183	0.61
Gender	13011	2942	0.00
Any secondary education	12394	2730	0.00
In relationship	4693	2818	0.10
CD4	−13	8	0.09
Physical health functioning	25	55	0.65

Model 2			

ART	−6144	4052	0.13
Time	1444	1969	0.46
ART × time	−218	2903	0.91
Change in physical health functioning	−8	66	0.91
Baseline covariates			
Age	91	183	0.62
Gender	13041	2955	0.00
Any secondary education	12400	2733	0.00
In relationship	4687	2820	0.10
CD4	−13	8	0.09
Physical health functioning	22	62	0.73
